# Supplementary material for: Mapping a genome-scale in vivo knockout screen to a mechanistic network model identifies VAV2, RASA1, and LEPR as regulators of cardiomyocyte hypertrophy
Source: bioRxiv. 2026 Sep 29:2026.09.24.754086. Preprint. [Version 1] doi: 10.64898/2026.09.24.754086 (PMC13648429; doi:10.64898/2026.09.24.754086)
Supplement: Supplementary file 1 [file NIHPP2026.09.24.754086v1-supplement-1.pdf]

bioRxiv preprint doi: https://doi.org/10.64898/2026.09.24.754086; this version posted September 29, 2026. The copyright holder for this preprint (which was not certified by peer review) is the author/funder, who has granted bioRxiv a license to display the preprint in perpetuity. It is made available under a CC-BY 4.0 International license.

Supplementary Figures

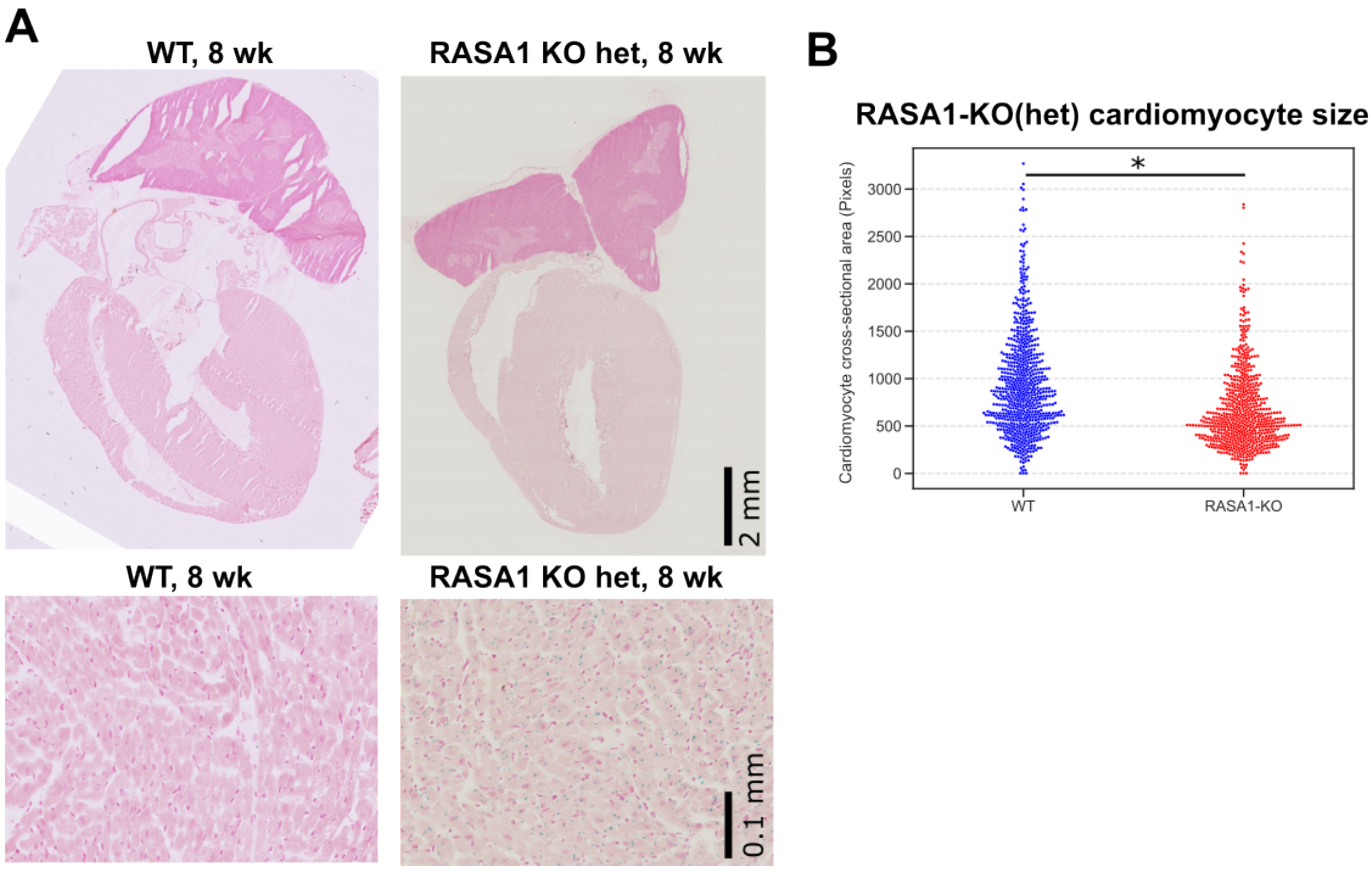

Supplementary Figure 1. Representative tissue sections of whole hearts and cardiomyocyte cross-sections from WT and RASA1-KO heterozygous mice at 8 weeks of age. F) Quantification of cardiomyocyte cross-sectional area from Figure 6D. *$p<0.05$ two-tailed T test with Sidak correction, n =744 WT myocytes and n=679 RASA1-KO myocytes.
